# Oral administration of N-acetyl cysteine prevents osteoarthritis development and progression in a rat model

**DOI:** 10.1038/s41598-019-55297-2

**Published:** 2019-12-10

**Authors:** Yosuke Kaneko, Nobuharu Tanigawa, Yuiko Sato, Tami Kobayashi, Satoshi Nakamura, Eri Ito, Tomoya Soma, Kana Miyamoto, Shu Kobayashi, Kengo Harato, Morio Matsumoto, Masaya Nakamura, Yasuo Niki, Takeshi Miyamoto

**Affiliations:** 1 Department of Orthopedic Surgery, Keio University School of Medicine, 35 Shinano-machi, Shinjuku-ku, Tokyo 160-8582 Japan; 2 Department of Advanced Therapy for Musculoskeletal Disorders II, Keio University School of Medicine, 35 Shinano-machi, Shinjuku-ku, Tokyo 160-8582 Japan; 3 Department of Musculoskeletal Reconstruction and Regeneration Surgery, Keio University School of Medicine, 35 Shinano-machi, Shinjuku-ku, Tokyo 160-8582 Japan; 4 Institute for Integrated Sports Medicine, Keio University School of Medicine, 35 Shinano-machi, Shinjuku-ku, Tokyo 160-8582 Japan; 5 Division of Oral and Maxillofacial Surgery, Department of Dentistry and Oral Surgery, Keio University School of Medicine, 35 Shinano-machi, Shinjuku-ku, Tokyo 160-8582 Japan; 6 Department of Orthopedic Surgery, Kumamoto University, 1-1-1 Honjo, Chuo-ku, Kumamoto 860-8556 Japan

**Keywords:** Experimental models of disease, Drug development

## Abstract

The number of osteoarthritis patients is increasing with the rise in the number of elderly people in developed countries. Osteoarthritis, which causes joint pain and deformity leading to loss of activities of daily living, is often treated surgically. Here we show that mechanical stress promotes accumulation of reactive oxygen species (ROS) in chondrocytes *in vivo*, resulting in chondrocyte apoptosis and leading to osteoarthritis development in a rat model. We demonstrate that mechanical stress induces ROS accumulation and inflammatory cytokine expression in cultured chondrocytes *in vitro* and that both are inhibited by treatment with the anti-oxidant N-acetyl cysteine (NAC). *In vivo*, osteoarthritis development in a rat osteoarthritis model was also significantly inhibited by oral administration of NAC. MMP13 expression and down-regulation of type II collagen in chondrocytes, both of which indicate osteoarthritis, as well as chondrocyte apoptosis in osteoarthritis rats were inhibited by NAC. Interestingly, osteoarthritis development in sham-operated control sides, likely due to disruption of normal weight-bearing activity on the control side, was also significantly inhibited by NAC. We conclude that osteoarthritis development in rats is significantly antagonized by oral NAC administration. Currently, no oral medication is available to prevent osteoarthritis development. Our work suggests that NAC may represent such a reagent and serve as osteoarthritis treatment.

## Introduction

Osteoarthritis is characterized by degradation of articular cartilage in joints, which causes joint pain and deformity. Currently, the number of osteoarthritis patients is dramatically increasing due to growth of the aging population. Treatment strategies depend on osteoarthritis stage, as assessed by radiographic imaging such as Kellgren-Lawrence (KL) system used to evaluate narrowing of joint space and osteophyte formation, and early stage (KL 1 and 2) patients are advised to exercise and reduce joint mechanical loading by weight loss. Patients with higher grade osteoarthritis (KL stages 3 and 4) are treated with NSAIDs or opioids, or occasionally by surgery, such as total knee arthroplasty. Thus, more expensive and invasive treatments are necessary to treat osteoarthritis that has progressed. Articular cartilage has limited regeneration capacity^1^. Thus preventing cartilage degradation is mandatory to block osteoarthritis progression.

Articular chondrocytes are derived from mesenchymal stem cells and produce various extracellular matrix proteins (ECMs) such as proteoglycans and type II collagen, and function in shock absorption and enabling smooth movement of joints. Mild articular cartilage damage can repair itself due to chondrocytes’ regenerative activities such as chondrocyte cloning regulated by reversion-inducing cysteine-rich protein with Kazal motifs (RECK)^2,3^ and semaphorin 3A^4–6^. However, if articular cartilage damage covers a large area or occurs continuously, repair is no longer possible due to the limited proliferation and repair capacity in chondrocytes, and damage becomes irreversible, leading to osteoarthritis.

ECM degradation, which characterizes osteoarthritis, is promoted by several protein-degrading enzymes, including matrix metalloproteinases (MMPs), particularly MMP3 and MMP13^7–10^, and members of the a disintegrin and metalloproteinase with thrombospondin motifs (ADAMTS) family such as ADAMTS4 and ADAMTS5^11–16^. Hypoxia inducible factor 2 (HIF2) also reportedly acts as an upstream regulator of osteoarthritis inducible factors^17–19^. Muscle mass decreases age-dependently in part due to decreasing levels of IGF-1^20^, which results in joint instability and subsequent osteoarthritis development in patients^21–23^. Joint instability established by surgical dissection of the anterior cruciate ligament (ACL) is commonly used as an animal model of mechanical stress-induced osteoarthritis^24^. Mechanical loading due to obesity is also a risk factor for osteoarthritis development^25,26^. Analysis of animal models indicates that activation of nuclear factor kappa B (NF-κB) caused by excessive mechanical loading also functions in osteoarthritis development^27^. Moreover, several inflammatory cytokines and reactive oxygen species (ROS) are known to play a role in osteoarthritis development^28,29^, however, pathological mechanisms underlying osteoarthritis development are not yet fully understood, slowing efforts to prevent osteoarthritis progression.

N-acetylcysteine (NAC), a glutathione precursor, reportedly acts as an anti-oxidant factor by targeting ROS^30^, and anti-oxidant effects of NAC in animal models exhibiting pathologies associated with ROS have been reported^31–33^. Currently, NAC is used clinically to treat acetaminophen poisoning but has not been approved to treat patients with osteoarthritis.

Here, we demonstrate that mechanical stress promotes ROS accumulation in chondrocytes *in vivo* and *in vitro*. We show in a cell culture system that mechanical stress induces tumor necrosis factor alpha (TNFα) expression, which is inhibited *in vitro* by a treatment chondrocytes with the anti-oxidant N-acetyl cysteine (NAC). Oral NAC administration also prevented osteoarthritis development and progression in an animal model. Overall, we propose that an oral administration of NAC represents an option to block osteoarthritis development and progression.

## Results

### Mechanical stress induces ROS accumulation in chondrocytes *in vitro*

**Figure 1:**
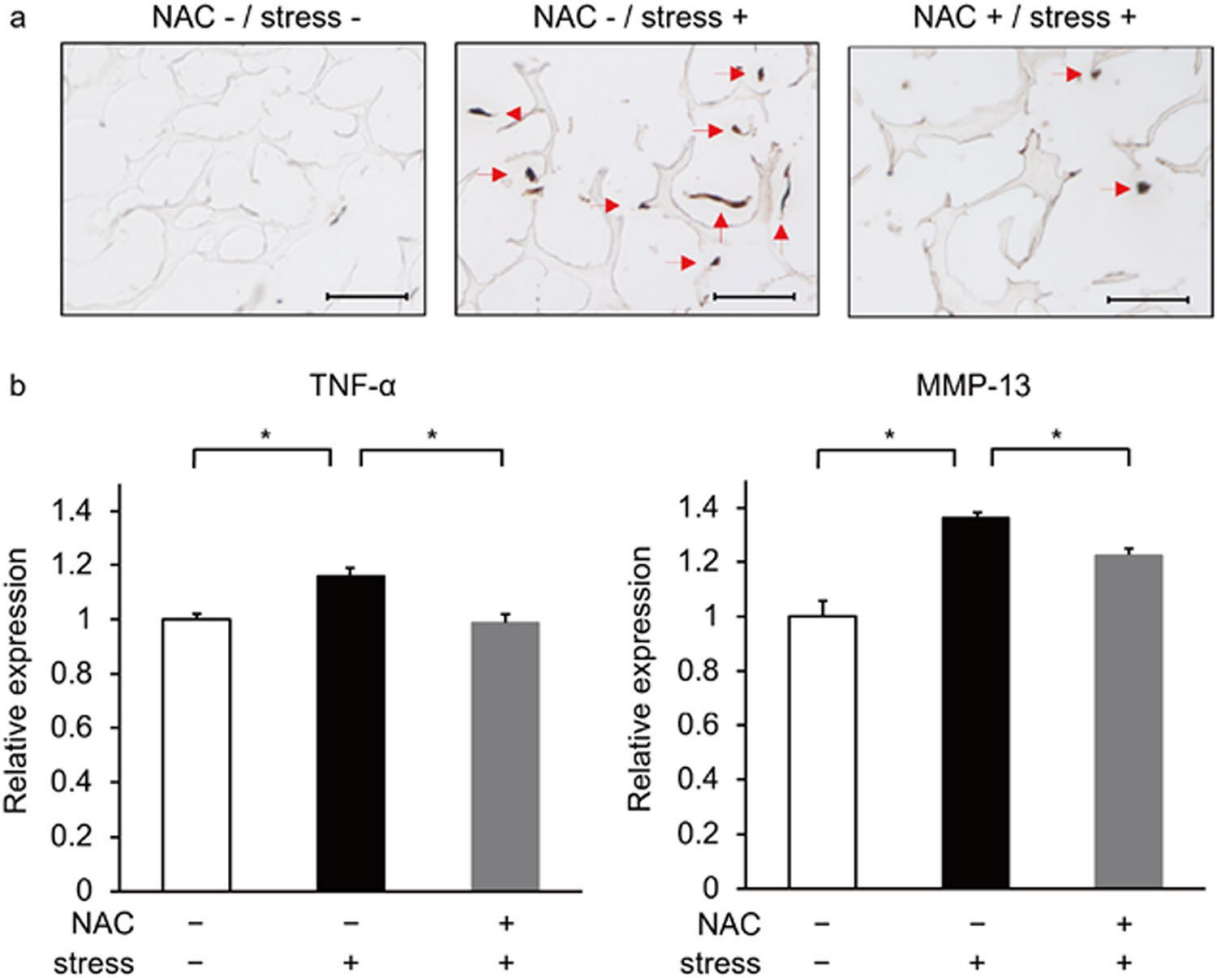
ROS accumulates in primary chondrocytes following mechanical stress. (**a**) Primary chondrocytes were isolated from ribs of 5-week-old wild-type male Wistar rats and cultured with or without cyclic loading stresses (40 g weight, 2 Hz) for 30 minutes. Cells were then stained with mouse-anti-8OHdG antibody followed by Alexa Fluor 488-conjugated goat anti-mouse IgG, treated with DAB and observed under a microscope. Bar, 100μm. (**b**) Primary chondrocytes similarly isolated were cultured with or without cyclic loading stresses (40 g weight, 2 Hz) in the presence or absence of NAC (10 µM) for 30 or 60 minutes and then evaluated for *TNFα* expression or *MMP-13*, respectively, by real-time PCR. Data represent mean *TNFα* expression or *MMP-13* expression relative to *β-actin* ± SD (n = 3 each, *P < 0.05). Representative data of at least two independent experiments are shown.

To assess effects of mechanical load in chondrocytes *in vitro*, we isolated rat rib or articular chondrocytes, cultured them with or without mechanical loading, and evaluated ROS accumulation by staining with 8OHdG, a ROS marker. Both ROS and TNFα are risk factors for osteoarthritis^34^. Chondrocytes cultured with mechanical loading exhibited ROS accumulation as well as *TNFα* induction (Figs. 1a,b and S1). Treatment with the anti-oxidant NAC blocked ROS accumulation in those chondrocytes (Fig. 1a). *TNFα* expression was also significantly blocked by treatment of cells with NAC (Figs. 1b and S1). Similarly, NAC treatment also blocked expression of *matrix metalloprotease 13* (*MMP13)*, which undergoes degradation in chondrocytes in osteoarthritis^33,35^, in chondrocytes subjected to mechanical loading (Figs. 1b and S1). These results suggest that mechanical stress promotes osteoarthritis development through several ROS-induced factors, and that osteoarthritis in those contexts can be ameliorated by NAC treatment.

### NAC administration blocks osteoarthritis development *in vivo*

**Figure 2:**
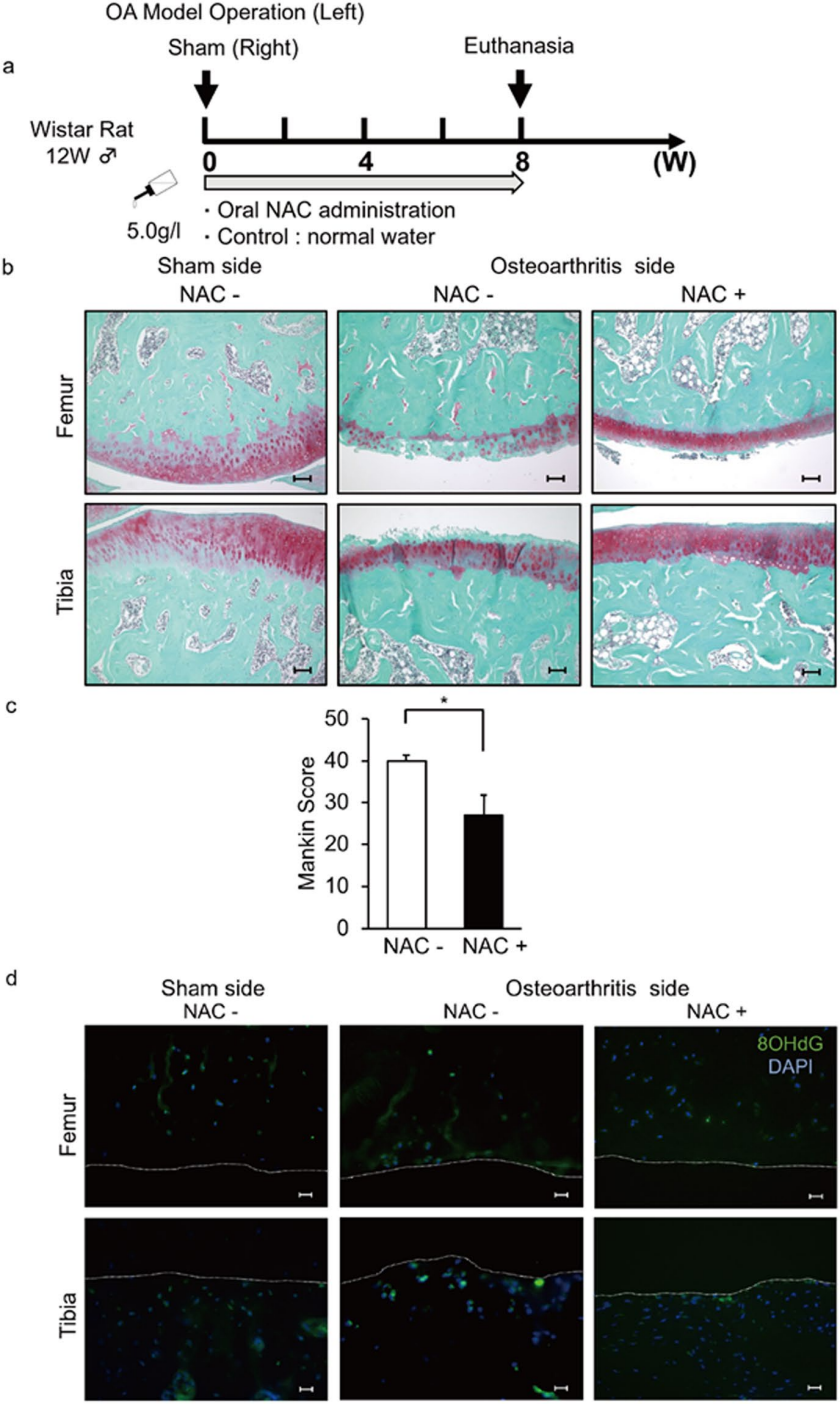
Oral NAC administration inhibits osteoarthritis development in rats. (**a**–**d**) (**a**) Schematic showing NAC treatment in the rat osteoarthritis model. Mechanical stress-induced osteoarthritis models were created in the left knee joints of 12-week-old wild-type male Wistar rats. Right sides were sham-operated. NAC (5.0 g/l) or control water was supplied for eight weeks (w) starting at the time of surgery. (**b**,**c**) Then, knee joint sections (femur and tibia) were prepared and stained with safranin O and the Mankin score was evaluated. Bar, 100μm. Data shows the mean total Mankin score ± SD (n = 3 each, *P < 0.05). (**d**) Surgery or sham-surgery was performed in left or right knee joints, respectively, of 12-week-old wild-type male Wistar rats, and rats were maintained with or without NAC water (5.0 g/l) for eight weeks afterwards. Shown are right sham operated and left osteoarthritis-operated knee joint sections prepared after the procedure and stained with mouse anti 8-OHdG followed by Alexa Fluor 488-conjugated goat anti-mouse IgG. Nuclei were visualized by DAPI. Bar, 20μm. Representative data of at least two independent experiments are shown.

We next created a mechanical stress-induced osteoarthritis model in rats by as described in the Methods section on the left side to generate knee joint instability (Fig. 2a). The right side was sham-operated and served as a control (Fig. 2a). Rats developed osteoarthritis on the left side, as evidenced by reduced staining in joint tissue of safranin O, which stains proteoglycans, and showed an elevated Mankin score, which also indicates arthritis development (Fig. 2b,c). ROS accumulation, as assessed by 8OHdG staining, was seen in joints showing osteoarthritis (Figs. 2d and S2); however, ROS accumulation was also detected on the sham-surgery sides (Fig. 2d).

Since most osteoarthritis patients are elderly, treatment periods are prolonged due to patients’ extended lifetime. Thus oral delivery of therapies could be more desirable as treatment than joint injection for patients undergoing long-term treatment. To test such a strategy, we administered NAC orally in drinking water to rats with osteoarthritis (Fig. 2). In control mice, NAC administration did not alter water or food intake nor did it change body weight as compared to mice administered water without NAC (Fig. S3a–d). Both reduced safranin O staining and the elevated Mankin score seen in osteoarthritis rats were significantly rescued by an oral NAC administration (Fig. 2b,c), and ROS accumulation in chondrocytes seen in osteoarthritis rats was also blocked by NAC treatment (Fig. 2d).

**Figure 3:**
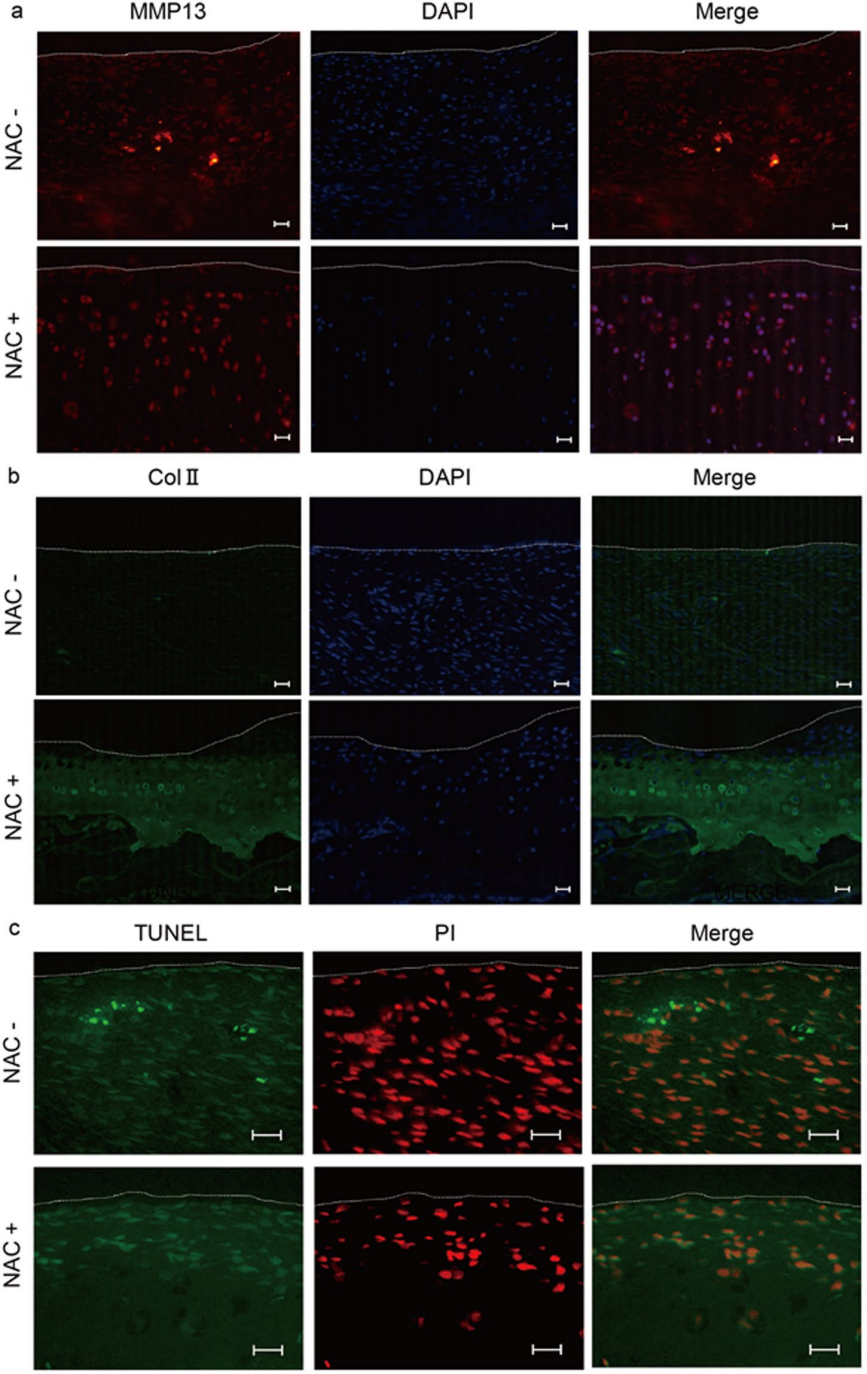
NAC treatment inhibits osteoarthritis development *in vivo*. (**a**–**c**) (**a**) Osteoarthritis or sham surgery was performed in left or right knee joints, respectively, in 12-week-old wild-type male Wistar rats, and rats were maintained with or without NAC water (5.0 g/l) for eight weeks thereafter. Left osteoarthritis knee joint sections were then prepared and stained with rabbit anti-rat MMP13 (**a**) or mouse anti-rat Col II (**b**) followed by Alexa Fluor 546-conjugated goat anti-rabbit IgG or Alexa Fluor 488-conjugated goat anti-mouse IgG, respectively. Nuclei were visualized by DAPI. Specimens were also stained with TUNEL, and nuclei were stained with propidium iodide (PI) (**c**), and observed under a fluorescence microscope. Bar, 20 μm. White dotted lines indicate joint surfaces of the tibia. Bar, 20 μm.

Expression of MMP13, a marker of osteoarthritis development, in chondrocytes *in vivo* was inhibited by oral NAC administration (Fig. 3a), and reduced type II collagen expression in articular cartilage was rescued by NAC treatment *in vivo* (Fig. 3b). Chondrocyte apoptosis, as determined by TUNEL staining, was induced in joint tissues in osteoarthritis rats but blocked by NAC treatment (Fig. 3c).

**Figure 4:**
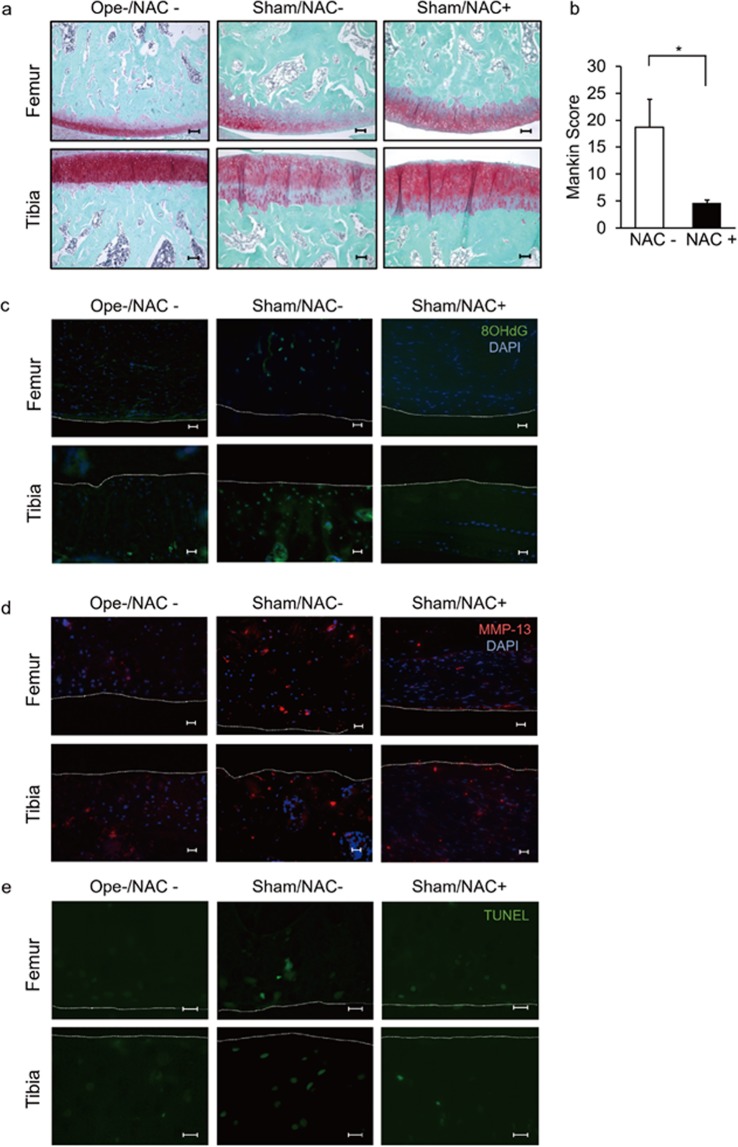
Oral NAC administration inhibits osteoarthritis development on the sham-operated side. Osteoarthritis or sham surgery was performed in the left or right knee joints, respectively, in 12-week-old wild-type male Wistar rats, and rats were maintained with or without NAC water (5.0 g/l) for eight weeks thereafter. Then, right knee joint sections (femur and tibia) were prepared and stained with Safranin O (**a**) and the mean total Mankin score was assessed (**b**). Data shows the mean Mankin score ± SD (n = 3 each, *P < 0.05). Bar, 100 μm. (**c**,**d**) The right knee sections were also stained with mouse anti 8-OHdG **(c)** or rabbit anti-rat MMP13 (**d**), followed by Alexa Fluor 488-conjugated goat anti-mouse IgG or Alexa Fluor 546-conjugated goat anti-rabbit IgG, respectively. Nuclei were visualized by DAPI. Specimens were also stained with TUNEL (**e**), and observed under a fluorescence microscope. Intact right knee joint sections of 20-week-old wild-type male Wister rat maintained with normal water was also prepared and stained with Safranin O, 8OHdG, MMP-13, TUNEL to compare with the sham surgery performed samples (a-e). White dotted lines indicate joint surfaces of the femur and tibia. Bar, 20 μm.

Sham surgery was performed on the right side of our osteoarthritis model, prompting those sides to support altered weight bearing (Fig. 4a,b). ROS accumulation was detected in the sham-operated sides but not in joints from the non-surgery group (Figs. 2d and 4c). We observed that osteoarthritis, based on *MMP13* expression and induction of apoptosis of chondrocytes, developed spontaneously even on the sham-operated sides (Fig. 4d,e). Moreover, osteoarthritis development on sham-operated sides was also significantly inhibited by NAC treatment (Fig. 4b).

### NAC administration effectively inhibits previously established osteoarthritis *in vivo*

**Figure 5:**
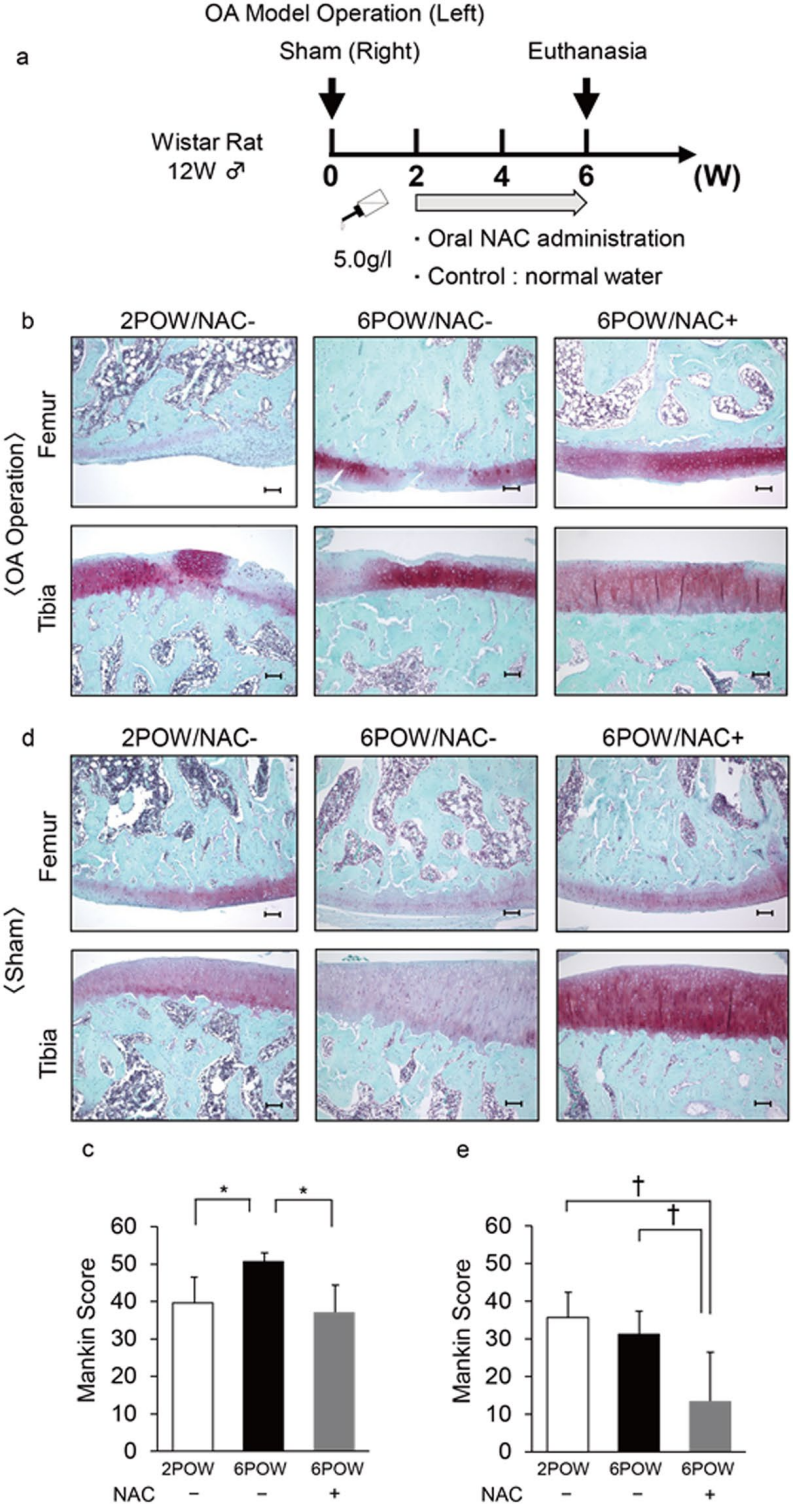
Oral NAC administration blocks progression of previously established osteoarthritis *in vivo*. (**a**) Schematic showing the protocol used to administer NAC orally starting at two weeks after surgery. (**b**–**e**) Osteoarthritis or sham surgery was performed in the left or right knee joints, respectively, in 12-week-old wild-type male Wistar rats, and rats were maintained with or without NAC water (5.0 g/l) from two weeks to six weeks after surgery. Two weeks after surgery (2POW) (and before NAC treatment) or six (6POW) weeks after surgery (when NAC had been administered four weeks), knee joint sections (femur and tibia) were prepared and stained with Safranin O, and the Mankin score was assessed. Bar, 100 μm. (**c**,**e**) Data shows the mean Mankin score ± SD (n = 3 each, *P < 0.05).

Since osteoarthritis patients often seek treatment for ongoing joint pain, we administered NAC to rats two weeks after surgery to evaluate whether oral NAC administration could ameliorate osteoarthritis already established (Fig. 5a). ROS accumulation was seen in the joints by two weeks after surgery (Fig. S2). Osteoarthritis development, as evaluated by reduced safranin O staining and elevated Mankin score, was induced by two weeks after surgery, at a time that we began NAC administration (Fig. 5b). As shown in Fig. 5c, oral NAC administration blocked osteoarthritis progression even after osteoarthritis was established (Fig. 5c). Oral NAC administration also effectively reduced arthritis development on the sham-operated side (Fig. 5d,e).

## Discussion

**Figure 6:**
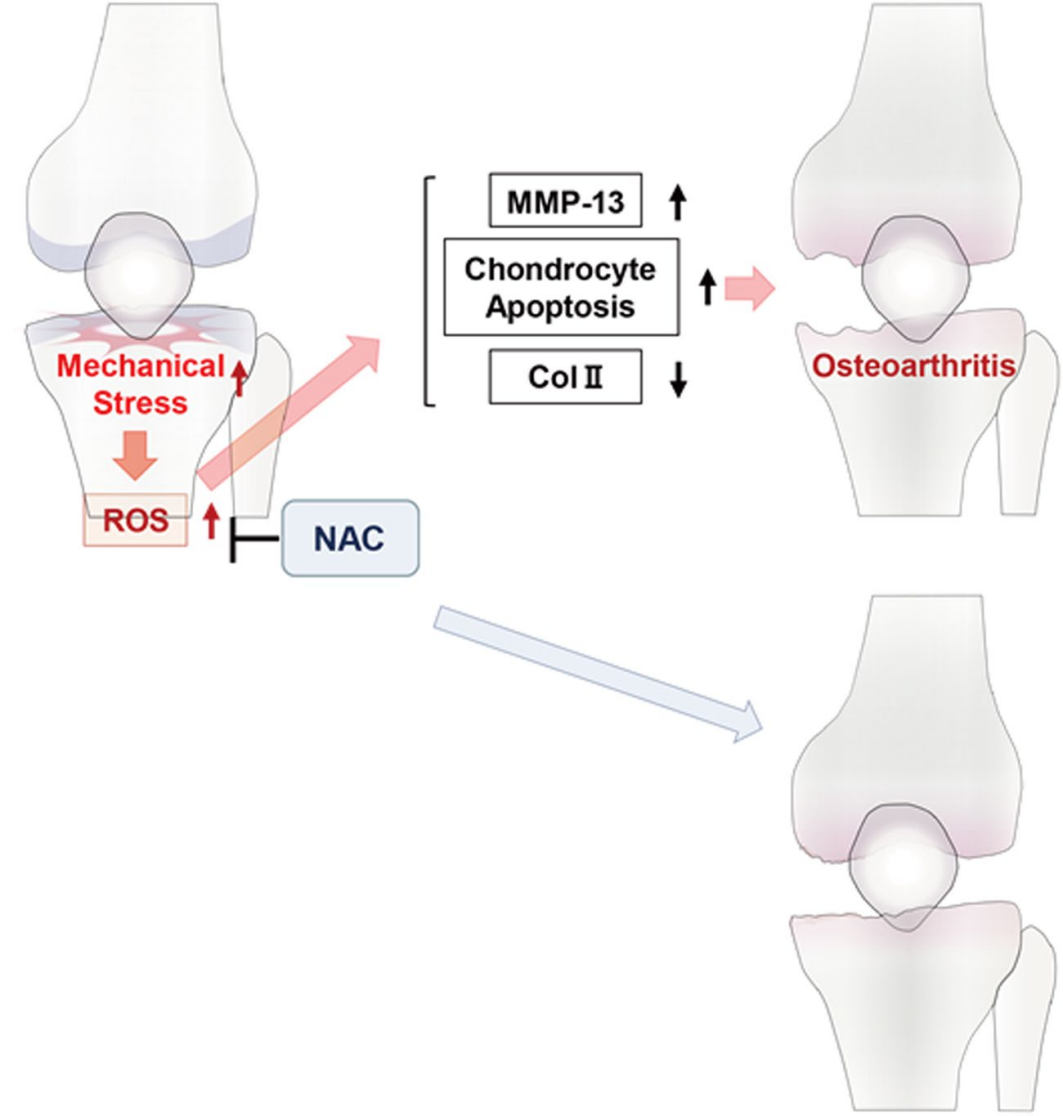
Schematic showing osteoarthritis development promoted by mechanical stress. Mechanical stress due to joint instability promotes ROS accumulation in articular cartilage, resulting in increased levels of TNFα and MMP13 and down-regulation of type II collagen. Articular cartilage then undergoes apoptosis, leading to osteoarthritis development.

Given that articular cartilage has limited regenerative potential, once osteoarthritis is established, it becomes irreversible^36,37^, making invasive therapy such as total knee arthroplasty. Some regenerative therapies are now available for chondrocytes but they are expensive. Since pain is the chief complaint of osteoarthritis patients, NSAIDs, steroids and opioid can relieve symptoms, but those agents do not block chondrocyte degeneration, and some patients undergo invasive treatment even after such treatments. Therefore, more important than pain relief, it is necessary to save chondrocytes from degeneration at early stages in order to block osteoarthritis development. Injection of hyaluronic acid into joints reportedly inhibits osteoarthritis development^38^, but protective oral agents are currently not available. Based on our study, we propose that orally-administered NAC is a candidate agent that might inhibit osteoarthritis development and progression (Fig. 6).

Molecular changes driving osteoarthritis development are known to resemble those during seen in endochondral ossification. In that process, proliferating chondrocytes predominantly express Sox9 and type II collagen and then differentiate into hypertrophic chondrocytes that express Runx2, MMP13 and type X collagen^39^. Hypertrophic chondrocytes also express vascular endothelial cell growth factor (VEGF)^40^, which promotes vascular invasion into the avascular chondrocyte region^41^. MMP13 degrades the ECM, and chondrocytes are replaced by osteoblasts^42^. ROS accumulates in prehypertrophic and hypertrophic chondrocytes and stimulates chondrocyte hypertrophy^33^. Similar to endochondral ossification, articular cartilage is also an avascular tissue and predominantly expresses Sox9 and type II collagen in normal circumstances but upregulates Runx2, HIF2, MMP13 and type X collagen as cartilage degenerates and osteoarthritis develops, in a pattern inverse to Sox9 and type II collagen expression^43^. Vascular invasion and ectopic ossification, including osteophyte formation, are seen in osteoarthritis patients^44^. Our findings suggest that anti-oxidant treatment could suppress such chondrocyte degeneration.

Excess ROS levels in proliferating chondrocytes alter normal endochondral ossification^33^. The disease Ataxia Telangiectasia Mutated (ATM) is characterized by excessive ROS accumulation in various cell types, among them, hematopoietic stem cells (HSCs) and proliferating chondrocytes^31–33,45^. Patients with ATM exhibit short life span and growth retardation due to ROS accumulation in these cells^46^. We and others previously reported that NAC administration rescued perturbed HSC and chondrocyte homeostasis, at least in part, in ATM animal models^31–33^. Thus, chondrocyte hypertrophy and degeneration during development and in osteoarthritis development, respectively, are both likely promoted at least in part by ROS, and can be blocked by treatment with the anti-oxidant NAC.

Intervertebral disc tissue is the largest avascular tissue in the body and is hypoxic^47^. Interestingly, VEGF functions in maintaining intervertebral disc homeostasis^47^. Analysis of VEGF-deficient mice also indicates that VEGF is required for chondrocyte survival during endochondral ossification^48^. In an intervertebral disc puncture model, which produces disc inflammation and degeneration, ROS reportedly accumulates in annulus fibrosus cells, and such degeneration is reportedly prevented by anti-oxidant treatment^49^. Thus, NAC administration may also be effective in inhibiting intervertebral disc degeneration brought on by mechanical loading.

Mechanical stress reportedly stimulates the glycoprotein lubricin, which is encoded by the *Prg4* gene and expressed in the superficial zone of articular cartilage^50^. At present, it is not clear whether NAC treatment can promote lubricin expression in cartilage, and further studies are needed to address this question. Nonetheless, we propose that NAC treatment is a potential therapy to block osteoarthritis development and progression.

## Methods

### Rats

All 12-week-old male Wistar rats were purchased from Japan Clea (Tokyo, Japan) and were placed under specific pathogen-free conditions in animal facilities certified by the Keio University Institutional Animal Care and Use Committee. Animals were housed up to 3 rats per cage and kept on a 12 h light/dark cycle. Water and food was available ad libitum, and whole rats were maintained in an environment in accordance with committee guidelines. All animal experimental protocols were approved by the Keio University Institutional Animal Care and Use Committee, and performed in accordance with the Guidelines certified by that committee.

### Animal model of knee osteoarthritis

All procedures were performed under anesthesia in 12-week-old male Wistar rats. After a median incision above the left patella, a para-patella approach was applied to reach intra-articular space in left knee joints. Subsequently, the medial meniscotibial ligament was excised and the bilateral menisci were removed. Then, the medial cruciate ligament (MCL), the ACL, and the lateral extensor tendon were dissected to generate instability in the left knee joint. Sham surgery was performed in the right knee joint of the same rats.

### Oral NAC administration

NAC (Sigma-Aldrich, St.Louis, MO, USA) was dissolved in distilled water (NAC water, 5.0 g/l), and normal or NAC-containing water was administered orally to rats for periods indicated in figure legends.

### Histopathology and fluorescent immunohistochemistry

The bilateral knee joints of Wistar rats were fixed in 10% neutral-buffered formalin, embedded in paraffin, and cut into 4-μm sections. Samples were decalcified in 10% EDTA, pH7.4, before embedding. Hematoxylin and Eosin (HE) or safranin-O staining was performed using standard procedures, and samples were observed under a microscope using the software supplied for BioRevo (BioRevo, Keyence, Tokyo, Japan). To evaluate degenerative changes in articular cartilage, the Mankin score was calculated as described^51,52^, and total Mankin score was collected from four different regions in the joints. For fluorescent immunohistochemistry, sections were subjected to 500 W microwaving for 10 minutes and immersed in 10 mM citrate buffer solution (pH 6.0) for antigen retrieval. After blocking with 3% BSA in PBS for 1 h, sections were stained with mouse anti-Col II (1:100 dilution, Abcam, UK), rabbit anti-MMP13 (1:100 dilution, Abcam, UK) or mouse anti-8OHdG (1:100 dilution; JaICA, Shizuoka, Japan) overnight at 4 °C. After 3 PBS washes, sections were stained with Alexa Fluor 488-conjugated goat anti-mouse IgG (1:100 dilution; Invitrogen, Waltham, MA, USA) for Col II or 8OHdG, or Alexa Fluor 546-conjugated goat anti-rabbit IgG (1:100 dilution; Invitrogen, Waltham, MA, USA) for MMP-13, for an hour at room temperature. DAPI (1:5000; Wako Pure Chemicals Industries, Osaka, Japan) was used as a nuclear stain.

### DAB or TUNEL staining

Staining was performed using standard procedures (ImmPRESS Reagent, Vector Laboratories, Burlingame, CA, USA, or TUNEL kits, R&D, Minneapolis, MN, USA).

### Primary chondrocyte culture

Knee articular cartilage or rib chondrocytes were dissected from 5-week-old male Wistar rats, minced, and treated with 0.25% trypsin-EDTA for 5–10 minutes (Gibco, Waltham, MA, USA). Samples were dissolved in a 0.1% collagenase solution in water. After centrifugation 5 min at 1000 rpm, supernatants were harvested. Debris was removed using a 70μm cell strainer, remaining material was centrifuged 5 min at 1,000 rpm, and chondrocytes were suspended in DMEM. Cyclic loading stresses (40 g weight, 2 Hz, 30 min) were applied to isolated chondrocytes by a Cyclic Load Stimulator CLS5J-Z/CLS50S-Z (TechnoView Inc, Osaka, Japan), with or without NAC (10 μM). *TNFα* and *MMP-13* expression in chondrocytes were determined by real-time PCR, and ROS accumulation was detected by immunohistochemistry.

### RNA isolation and real-time PCR

Total RNA was isolated from cultured chondrocytes using TRIzol reagent (Invitrogen, Waltham, MA, USA) following general procedures. cDNA synthesis was then performed using oligo (dT) primers and reverse transcriptase (Wako Pure Chemicals Industries, Osaka, Japan). Quantitative real-time PCR was performed using a SYBR Premix reagent and a DICE Thermal cycler (Takara Bio Inc, Shiga, Japan). *β-actin* (*Actb*) expression served as an internal control. Primer sequences used for real-time PCR were as follows.

*β-actin*-forward: 5′-TCCTCCCTGGAGAAGAGCTATG-3′

*β-actin*-reverse: 5′-TGCCACAGGATTCCATACCCAG-3′

*TNFα*-forward: 5′-TCGGTCCCAACAAGGAGGAGAA-3′

*TNFα*-reverse: 5′- CTTGGTGGTTTGCTACGACGTG-3′

*MMP-13*-forward: 5′-AGACAGATTCTTCTGGCGTCTGC-3′

*MMP-13*-reverse: 5′-CTCGGGATGGATGCTCGTATGC-3′

### Statistical analysis

All results are expressed as means ± SD. Statistical significance of differences between groups was evaluated by Student’s t-test. *P* values < 0.05 were determined statistically significant.

## Supplementary information

Supplementary Figures and Legends
