# Supplementary material for: Oral administration of N-acetyl cysteine prevents osteoarthritis development and progression in a rat model
Source: Sci Rep. 2019 Dec 10;9:18741. doi: 10.1038/s41598-019-55297-2 (PMC6904562; doi:10.1038/s41598-019-55297-2)
Supplement: Supplementary file 1 — Supplementary Figures and Legends [file 41598_2019_55297_MOESM1_ESM.pdf]

Oral administration of N-acetyl cysteine prevents osteoarthritis development and progression in a rat model

Yosuke Kaneko[1], Nobuharu Tanigawa[1], Yuiko Sato[1,2], Tami Kobayashi[1,3], Satoshi Nakamura[1], Eri Ito[4], Tomoya Soma[5], Kana Miyamoto[1], Shu Kobayashi[1], Kengo Harato[1], Morio Matsumoto[1], Masaya Nakamura[1], Yasuo Niki[1,*] and Takeshi Miyamoto[1,2,3,6,*]

[1]Department of Orthopedic Surgery, [2]Department of Advanced Therapy for Musculoskeletal Disorders II, [3]Department of Musculoskeletal Reconstruction and Regeneration Surgery, [4]Institute for Integrated Sports Medicine, [5]Division of Oral and Maxillofacial Surgery, Department of Dentistry and Oral Surgery, Keio University School of Medicine, 35 Shinano-machi, Shinjuku-ku, Tokyo 160-8582, Japan, [6]Department of Orthopedic Surgery, Kumamoto University, 1-1-1 Honjo, Chuo-ku, Kumamoto 860-8556, Japan

Supplementary Figure Legends.

Figure S1. ROS accumulates in primary chondrocytes following mechanical stress.

Primary chondrocytes isolated from articular cartilage of 5-week-old wild-type male Wistar rats were cultured with or without cyclic loading stresses (40g weight, 2Hz) in the presence or absence of NAC (10μM) for 45 or 60 minutes and then evaluated for *TNFα* expression or *MMP-13*, respectively, by real-time PCR. Data represent mean *TNFα* expression or *MMP-13* expression relative to *β-actin* ± SD (n = 3 each, *P<0.05). Representative data of at least two independent experiments are shown.

Figure S2. ROS accumulates in the joints by two weeks after surgery.

Osteoarthritis or sham surgery was performed in left and right knee joints, respectively, in 12-week-old wild-type male Wistar rats, and they were maintained for two weeks afterwards. Left osteoarthritis-operated knee joint sections (femur and tibia) and right

sham-operated knee joint sections (femur and tibia) were prepared and stained with mouse anti 8-OHdG followed by Alexa Fluor 488-conjugated goat anti-mouse IgG. Nuclei were visualized by DAPI. Bar, 20μm. Representative data of at least two independent experiments are shown. White dotted lines indicate joint surfaces of the tibia.

Figure S3. Oral NAC administration does not alter water or food intake or body weight in Wistar rats. (**a** - **d**) Osteoarthritis or sham surgery was performed in the left or right knee joints, respectively, in 12-week-old wild-type male Wistar rats, and rats were maintained with or without NAC water (5.0g/l) for four (**a**, **b** and **c**) or eight (**d**) weeks after surgery. Amounts of NAC or normal water or food eaten by rats per day were then measured (**a** and **b**). Data represent mean amount of water or food ingested by rats per day ± SD (n=3 each, NS not significant). (**c** and **d**) Body weight evaluated at 0, 4 or 8 weeks after surgery in rats administered water with or without NAC. Data

represent mean body weight ± SD (n=6 each, NS not significant).

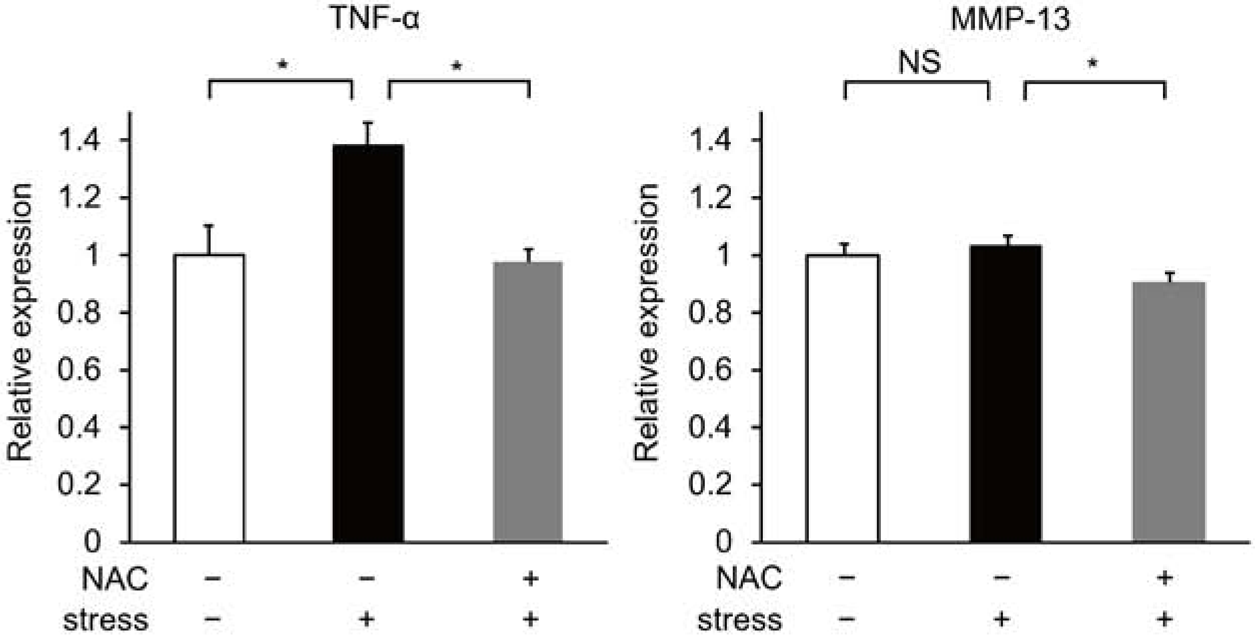
TNF-α
*
*
Relative expression
1.4
1.2
1
0.8
0.6
0.4
0.2
0
NAC – – +
stress – + +
MMP-13
NS
*
Relative expression
1.4
1.2
1
0.8
0.6
0.4
0.2
0
NAC – – +
stress – + +

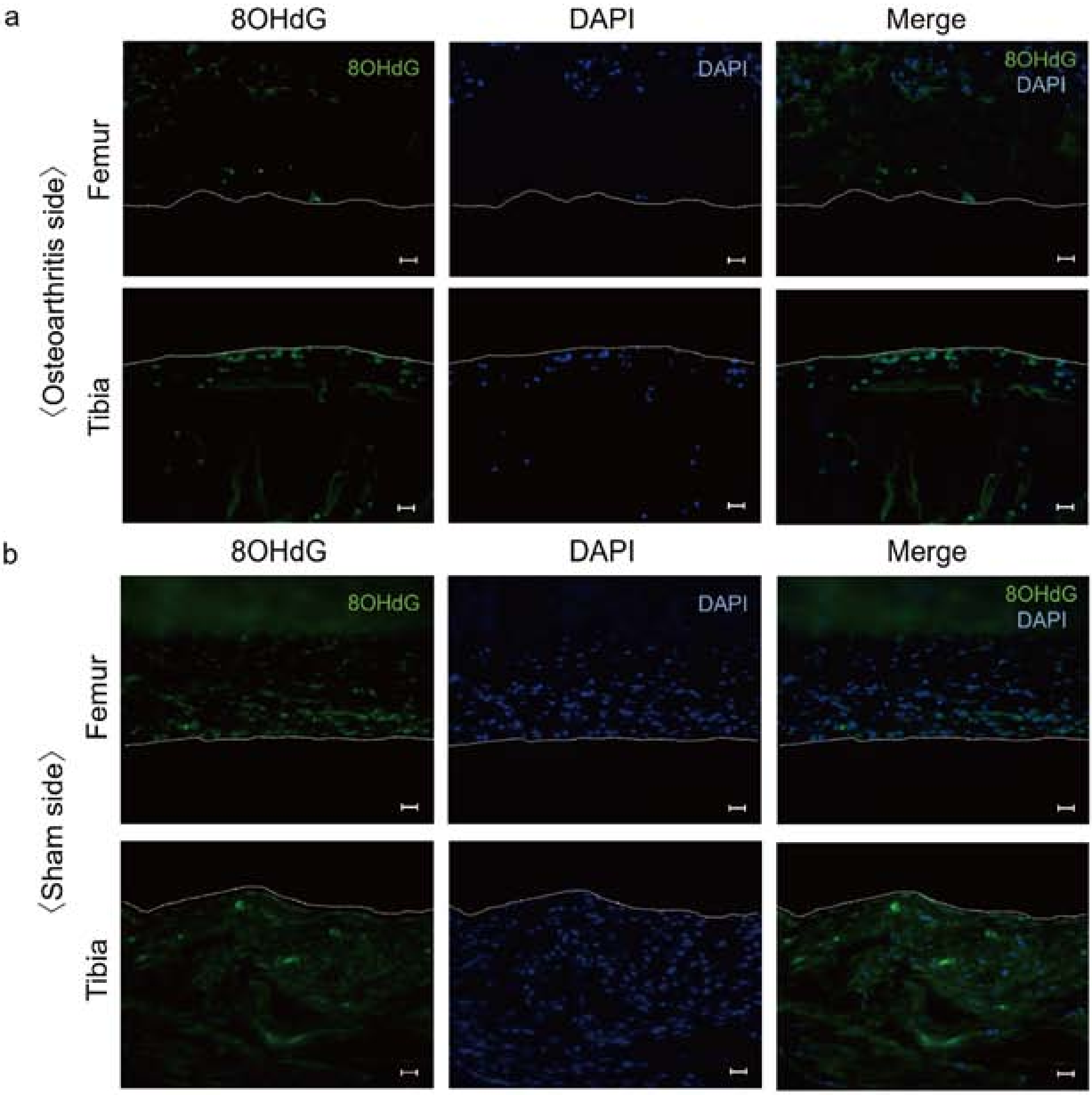
a
8OHdG
DAPI
Merge
〈Osteoarthritis side〉
Femur
Tibia
b
8OHdG
DAPI
Merge
〈Sham side〉
Femur
Tibia

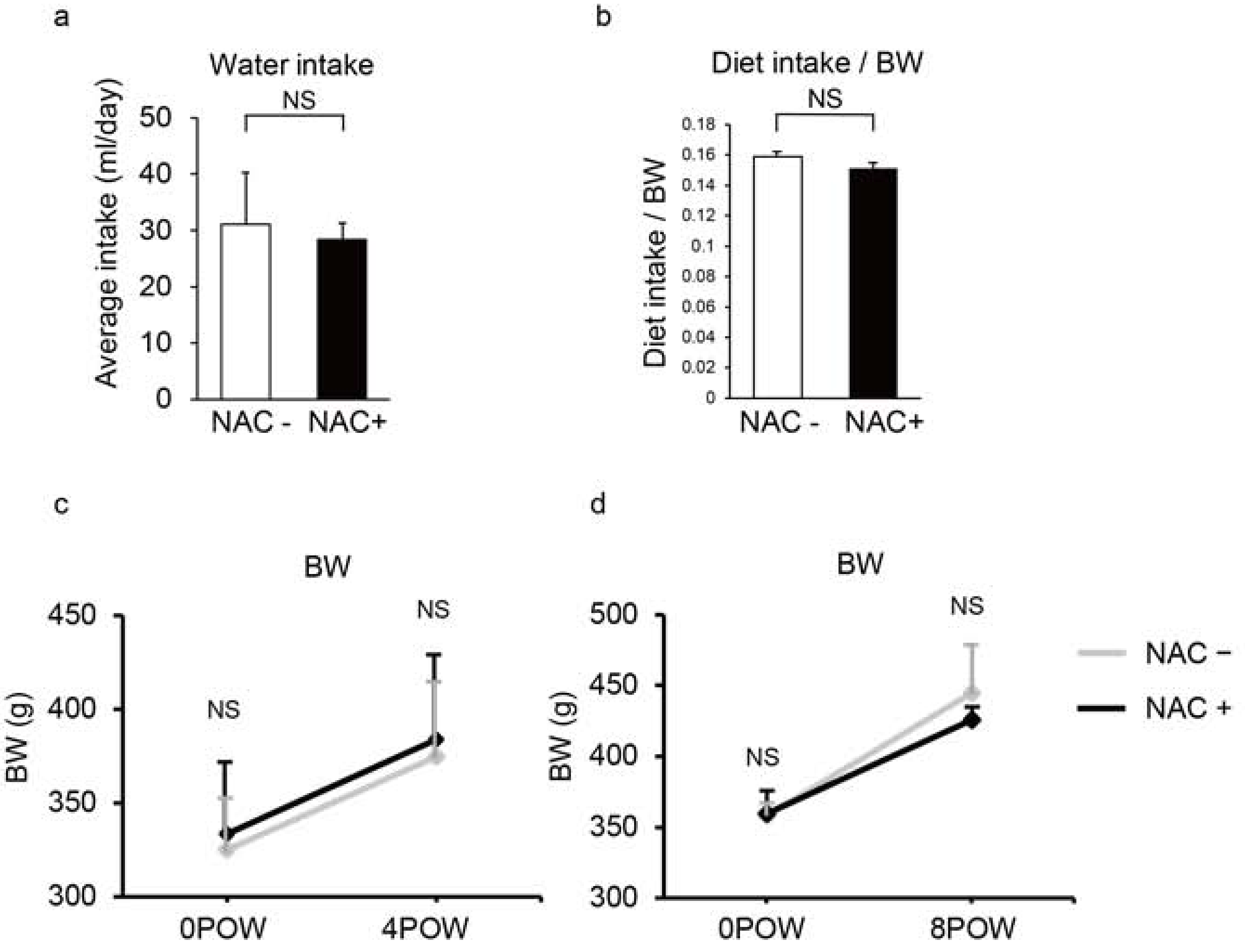
a
Water intake
NS
Average intake (ml/day)
50
40
30
20
10
0
NAC -
NAC+
b
Diet intake / BW
NS
Diet intake / BW
0.18
0.16
0.14
0.12
0.1
0.08
0.06
0.04
0.02
0
NAC -
NAC+
c
BW
NS
NS
BW (g)
450
400
350
300
0POW
4POW
d
BW
NS
NS
BW (g)
500
450
400
350
300
0POW
8POW
NAC −
NAC +
